# On Diseases of the Skin

**Published:** 1847-01-01

**Authors:** 


On Diseases of the Skin. By *Erasmus Wilson*, F.R.S., Consulting Surgeon to the St. Pancras Infirmary, Lecturer on Anatomy and Physiology in the Middlesex Hospital, &c. Second Edition, with coloured Plates, 8vo, pp. 482. London: Churchill, 1847.

Three years have scarcely elapsed since we had occasion to speak of this work in terms of commendation, and we are rejoiced to find that the opinion which we then gave of its merits has been participated in by the profession, for whom it was written. The first edition has now been many months out of print, and the present volume, with copious additions, and eight beautifully executed illustrations, is intended to supply its place. The rapid sale of the first edition has shown that the subject is one of much interest to the profession, and it proves also that the author has accomplished his task ably and satisfactorily. In truth, "the Diseases of the Skin," by Mr. Erasmus Wilson, may now be regarded as the standard work in that department of medical literature. The plates by which this edition is accompanied leave nothing to be desired, so far as excellence of delineation and perfect accuracy of illustration are concerned. They are drawn and coloured after nature, and are engraved on steel by Adlard, with a view to giving full effect to the peculiar tints of colour of these diseases. As an explanation of the nature and objects of the plates, we cannot do better than quote the author's words, and express our conviction that they completely fulfil his intentions.

"The present edition has the advantage over its predecessor of being illustrated with coloured delineations drawn from nature, and engraved on steel. The plates are eight in number, each representing a group of diseases. For example, if the reader wish to place before his eyes the group of 'congestive diseases' of the derma, he may turn to Plate 1, where he will find displayed, Urticaria, Roseola, and Erythema; Erysipelas being omitted, partly because its illustration is less necessary than that of other cutaneous diseases, and partly on account of the large extent of surface its delineation would require. If the reader would contrast the 'congestive group' of cutaneous diseases with others, he will find in Plate 2, the 'asthenic effusive group,' namely, Pemphigus and Rupia; in Plate 3, the 'sthenic effusive group,' namely, Herpes and Eczema; in Plate 4, the 'pustular group,' Impetigo and Ecthyma; in Plate 5, the 'papular group,' Lichen, Strophulus, and Prurigo; in Plate 6, the 'squamous group,' Lepra, Psoriasis, and Pityriasis; Plate 7 illustrates the peculiar morbid alteration of the skin termed Lupus non exedens; and Plate 8, certain diseases of the hair-follicles and hairs, namely, Acne, Sycosis, Favus, and Trichosis furfuracea.

"Another feature in the Plates which accompany this volume, and one at which I have especially aimed, is that of bringing together as many of the varieties of a given disease as possible. My reasons are twofold—firstly, that the leading characters of these varieties may be the more easily comprehended and contrasted; and, secondly, that the reader may be placed in possession of the largest amount of illustration admitting of being compressed into a limited space. For example, in the upper division of Plate 1 will be found four varieties of Urticaria, four varieties of Roseola, and, in the lower, six varieties of Erythema; making in the single plate fourteen specimens of cutaneous disease, the separate figures not being in any way stinted in dimensions, but being, in reality, of the size of life. With the most ample space at my command, I could have done little more than this. I might have repeated forms, but the matter to be impressed on the mind could not have been rendered more clear or more precise. Plate 2 represents, in the upper division, two varieties of Pemphigus; and, in the lower, three varieties of Rupia. The upper division of Plate 3 exhibits four varieties of Herpes, and the lower, the same number of varieties of Eczema. In Plate 4, the number of varieties of Impetigo, in the upper division, is four; and in the lower division, the varieties of Ecthyma are three. In Plate 5, there are, in the upper division, six varieties of Lichen; and in the lower, four varieties of Strophulus, and one of Prurigo. Plate 6 contains, in the upper division, three varieties of Lepra; and in the lower, three varieties of Psoriasis, and one of Pityriasis. Plate 7 is devoted to the single subject of Lupus non exedens; while in Plate 8 are four varieties of Acne, one of Sycosis, two of Favus, and one of Trichosis. So that, in the limited compass of eight octavo plates, no less than sixty-one subjects are represented." P. x.

In the present edition, Mr. Wilson has added very considerably to our stock of knowledge on the structure of the skin, the foundation upon which all our notions of pathology and all our practical applications must be based. "Conceiving," he observes, "that no real improvement could be made in the practical department of the subject in any other way than through the advancement of the scientific portion of these diseases, I have continued to bestow much attention and labour on the examination of the cutaneous tissues." In furtherance of this purpose, the author has introduced into the preliminary chapter on the Anatomy and Physiology of the Skin, his original researches on the structure of the epiderma, the hair, and several of the morbid tissues. The following is Mr. Wilson's description of the structure of a hair.

"In structure a hair is composed of three different tissues, namely, of a loose cellulated tissue which occupies its centre, and constitutes the medulla or pith; a fibrous tissue which incloses the preceding and forms the chief bulk of human hair; and a thin layer of superimposed scales which envelopes the fibrous structure, and forms the smooth, external surface of the hair.

"The medulla is absent in the minute or downy hairs, and is not unfrequently absent or small in quantity in fine hairs, from whatever region they are selected. In the coarser hairs of the head and of the body, on the other hand, it is always present, and it is especially remarkable in grey hair. It varies in breadth from a mere line to a cylindrical body of one-third the diameter of the hair, and is composed of large nucleated cells of a globular or oval figure filled with granules and packed together, apparently without order. When newly formed, these cells with their granules are distended with fluid, but in the shaft of the hair the cells frequently contain air, which from its highly refractive powers, gives the medulla a dark appearance when examined with the microscope. Varieties in structure of the hair are very unusual: I have, however, once observed the presence of two medullæ; the displacement of the medulla nearer to one side of the periphery of the hair than to the other in the short and thick hairs of the body is not so uncommon.

"The middle or fibrous layer of the hair is composed of oval-shaped cells closely packed together, and arranged in a linear order. These cells are identical in structure with the cells of the deep stratum of the epiderma, that is to say, they are composed of granules congregated around a central granule which constitutes the nucleus of the cell. When examined with the microscope it is not in all cases easy to discover the cells, but their component granules are always obvious, and from the plan of disposition of the cells, and their oblong shape, the granules have a linear arrangement, and assume the appearance of fibres. The hair-fibres offer some variety of appearance according to the focus in which they are viewed. For example, with a superficial focus the peripheral granules are alone seen, and the hair appears to be entirely composed of granules arranged in single rows. With a deeper focus the rows of granules appear to be associated in pairs, each pair having between them an unconnected row of dark and apparently nuclear granules. In this view the fibres resemble, very closely, a chain composed of open links. While with a still deeper focus, the centre of the cell, with its nucleus and granular periphery, is brought into view. In different hairs, these three appearances are seen with various degrees of distinctness.

"The colour of hair appears to reside partly in the granules, and partly in an intergranular pigmentary substance which occupies the interstices of the granules and of the fibres. The most deeply coloured granules are those which constitute the nuclei of the cells, and in the lighter hairs these alone give colour to the fibrous structure. In the darker hairs more or less of the peripheral granules are also coloured, and pigment may be observed in greater or less abundance in the interfibrous spaces. With respect to the granules the pigment appears to occupy their periphery, sometimes surrounding them completely, and sometimes occupying a portion only of their surface. In the peripheral granules of the cells the outer segment is the more frequent seat of the pigment, while many are entirely destitute of that production. This total absence of colour in many of the granules composing even the blackest hair, gives to the fibrous structure, when examined with the microscope, an interruptedly streaked appearance, and the irregular intermixture of pigment granules with colourless granules, bestows upon the tissue between the streaks a dotted character. In red hair, the granules have a delicate golden yellow tint, while the pigmentary matter is amber coloured. In the white hair of Albinoes and of the aged, the pigment is entirely wanting.

"The external layer of a hair is a thin and transparent envelope, measuring in the hairs of the head about $\frac{1}{8000}$ of an inch in diameter. It is composed of flattened scales similar to those of the epiderma, and the scales forming the surface of the layer overlap each other from the root to the point of the hair. The overlapping border of the scale is notched and convex, and forms a slight projection beyond the level of the surface. Seen with the microscope, the prominent edges of the scales have the appearance of undulating and jagged lines, which cross at right angles the shaft of the hair. The prominence of the edges of the superficial scales of a hair is the cause of the sensation of roughness which we experience in drawing a hair between the fingers from the point towards the root, a sensation which is not perceived when the direction of the hair is reversed. It explains also, the circumstance of hairs occasionally working their way into wounds, beneath the nails, and into the gums. In the hairs of the axilla the external layer is generally more or less split up into fibres, which give it a shaggy appearance. Sometimes this appearance occurs only on one side of the hair, or more on one side than the other, while at others it is equally conspicuous around the entire shaft. It forms a remarkable distinctive character of the hairs of this region, and is due, as I believe, not to original formation, but to their saturation with the perspiratory fluid." P. 25.

Again, in reference to the follicle of the hair, and the mode of implantation of the latter within the skin, he observes :—

"The follicle of the hair is a tubular canal excavated in the substance of the derma and lined by a thick layer of epiderma. It consequently presents the same three structures that enter into the composition of the skin, namely, an epidermal lining or sheath, a vascular layer, and the common fibrous tissue of the corium. Of the latter it is unnecessary to say more than that it offers the same characters around the hair as upon the surface of the derma, and that it sends a delicate sheath downwards upon the root of the hair when the latter extends into the subcutaneous areolar tissue. The vascular layer corresponds with the papillary layer of the derma, and supports a fine net-work of capillary vessels, which supply nutrition to the epidermal sheath and hair. The epidermal layer is composed of strata of superimposed cells, identical in structure with those of the epiderma. It is nearly as thick, and often thicker than the hair which it incloses, and lies in close contact with the latter, and, at its lower part it terminates in a slightly expanded and cellular mass, the pulp of the hair.

"The hair-follicle terminates inferiorly in a slightly dilated cœcal pouch, which is filled for about the extent of $\frac{1}{100}$ of an inch, with a mass of minute granules and cells. This mass of granules and cells is the pulp of the hair, and the cells are progressively converted into the substance of the hair. The cells produced at the middle of the fundus of the cœcal pouch necessarily proceed upwards in a direct line and are the first converted into fibres, hence the pointed character of a hair torn up from its root. The cells from the sides of the pouch proceed, with a gentle curve upwards and inwards, and merge into the substance of the root of the hair, and those from the upper part of the pulp assume an almost vertical position, and constitute, on the one hand, the outer layer of the hair, and on the other, the epiderma of the follicle. So that, at its upper part, the hair-pulp may be said to divide into two parts, a central and isolated part which constitutes the shaft of the hair, and a tubular sheath which remains in connexion with the vascular part of the follicle on the one hand, and is in apposition with the surface of the hair on the other. The structure of the pulp and the mode of growth of the hair remind us forcibly of the formation and growth of the teeth, and furnish an additional reason for regarding the latter as dermal appendages. They explain also the well-known fact that, if the epiderma be withdrawn from the derma when loosened by decomposition, the hairs may frequently be removed inclosed in their epidermal sheaths, which obviously extend uninjured around the bulb, and isolate the hair from the vascular part of the skin. I have found the vibrissæ nasi the best fitted for illustrating this point, and I may remark that the proof of such an organization completely sets at rest the question of the vascularity of the bulb." P. 27.

Among the subjects contained in this edition which have been retouched by the author, we observe the following in reference to the pathological nature of the papular diseases.

"In the preceding edition of this work, I stated my belief that the precise element of the dermal system affected in the papular diseases was the papillæ of the skin. More recent and careful examinations have proved to me that this is not the case, but that the real seat of morbid change is the vascular boundary of the various excretory tubules of the skin: for example, the sudoriferous and sebiferous ducts and hair-follicles. This fact being determined, we have an explanation of various of the phenomena which accompany the eruption: for example, the frequent perforation of the pimples by a hair, the formation of a thin scale upon the summit of the papula, the occasional appearance of a minute aperture in this situation, and the oozing of a transparent and colourless fluid from the same point. We can also better understand the provoking itching which is a symptom of the eruption, the obstruction which is offered to the escape of secretions, and the obstinacy of these disorders. The papulæ of prurigo are perfectly identical with the papulæ of lichen, the difference between them being, that the latter are generally acute in their course, while the former are always chronic. But there is an appearance of the skin in prurigo that must be familiar to all who are conversant with cutaneous diseases; an unevenness of surface, produced by numberless slight but broad elevations, separated from each other by the linear markings of the skin. Now these are the elevations which have been described by all dermatologists, not excluding myself, under the name of the broad and flat papulæ of prurigo. 'Soft and smooth papulæ, somewhat larger and less acuminated than those of lichen, and seldom appearing red or inflamed, except from violent friction. Hence an inattentive observer may overlook the papulæ altogether.' Rayer speaks of them as being 'soft to the touch, and broader than those of lichen, from which they also differ in preserving the natural colour of the skin.' 'They occasionally project in so slight a degree, that they appear to be situated rather in the substance than on the surface of the skin.' Now there is an evident obscurity about these descriptions, a contradiction in fact, which must have involved many in perplexity with regard to the real meaning of the authors. Papulæ, precisely defined, broad, soft, smooth and large, and yet, not distinguishable in colour from the adjacent skin, easily overlooked, and suggesting to the practised eye some uncertainty as to whether they were *in* or *upon* the skin. I will endeavour to explain the mystery.

"Prurigo, I believe to be, in its origin, a disease of the nervous system, and specially, of the cutaneous nerves. As a consequence of the altered innervation of the skin, the dermal tissues become changed in structure—namely, condensed and thickened. The most careless examination is sufficient to establish these two points; the skin feels hard, it moves like a piece of thick leather; the areæ included between the lines of motion are large; its natural suppleness is gone; its very colour is changed; it looks yellowish and dirty. But it is smooth; there are no such projections as we should call pimples, or if there be, they are few and scattered. Arrived at this point, there remains but one conclusion for the student. There are no papulæ, therefore the disorder cannot be prurigo. And yet the disease is so characteristically prurigo, that, setting aside the symptom of pruritus, the dermatologist is able to decide at once upon its name.

"What then, are the signs by which prurigo is so immediately distinguished? They are, the thickening and condensation of the skin, and the consequences of this condition. Upon close examination, the angular areæ included by the linear markings of the skin, are seen to be elevated above their natural level, the elevation being occasioned by the thickening of the derma. That this is the case is evident from the position of the pores—namely, in the furrows which constitute the linear markings, and at the point of divergence of several of these. The elevations, therefore, are simply the effect of a swollen state of the derma, the areæ being magnified by hypertrophy, and the linear markings being magnified in depth by the same cause. These swollen areæ are the so-called papulæ, the broad and flat and smooth papulæ. It is not, then, to be wondered at, that they should be with difficulty discerned, that they should be 'overlooked,' seeing, as I have shown, that they are not papulæ at all.

"But we do meet with papulæ in prurigo, although not a necessary feature of that disease. These papulæ are not the areæ of the linear markings of the skin; they occupy the grooves of the linear markings. They are, in fact, the pores raised into pimples, and are identical with the pimples of lichen. It is these latter which generally suffer abrasion of their tips from scratching, and then become surmounted by a small dark-coloured scab." P. 239.

The Chapter on the tuberculous affections of the skin, including the two forms of lupus, namely, non-exedens and exedens, is entirely new. Mr. Wilson is of opinion that the diseases severally denominated "Vitiligo" and "Leuce" are in reality one and the same morbid affection, and that they correspond in every particular with lupus non-exedens. The treatment from which he has obtained the best results in the cutaneous variety of lupus, is a prolonged course of the liquor hydriodatis hydrargyri et arsenici, at the same time modifying the local disease by the occasional application of acetum cantharidis.

Mr. Wilson's classification, which is essentially physiological and practical, we gave in full in our review of the first edition of this work. In the present, some trifling changes have been made, which have for their object to render the classification more complete; for example, Ichthyosis and Cornua are removed from the group of diseases depending on Hypertrophy of the papillæ to that of diseases of the sebiparous system, to which they more properly belong. The diseases of the sudoriparous system are more fully treated of under the heads of Idrosis, Anidrosis, Osmidrosis, Chromidrosis, and Hæmidrosis. Several newly-described forms of disorder of the sebiparous system are considered under the names of Xeroderma, Stearrhœa, and Narcosis folliculorum. And Ringworm and Plica are grouped under the same generic name, both diseases presenting, according to Mr. Wilson, the same elementary pathological change. Thus we read :—

"Two diseases only come strictly under this denomination, as being characterised by a morbid alteration in the structure of the hair. One is amongst the most common of the diseases of the scalp of this country, namely, ring-worm; the other is a disease of central Europe, and particularly of the marshy districts of Poland, the plica polonica. Much confusion has existed with regard to the former of these affections, in consequence of the variety of names which have been assigned to it, and also from the fact of the generic title comprehending diseases of a totally different character. Moreover, the names themselves are ill chosen, the term tinea relating to the condition of the hair at a period when the disease has been in existence for some time; while the term porrigo was selected by Willan, only because it had been in use among the ancient classic writers; neither of these terms having any reference to the nature of the disease. Under these circumstances, I consider that a first step to the proper understanding of this affection, and the removal of existing difficulties, might be made by adopting for its designation the term *trichosis,* proposed by Dr. Mason Good. I am further induced to give a preference to this term by finding it to coincide with what I believe to be the true pathological nature of the disease, namely, a morbid action producing the degeneration and destruction of the hairs." P. 415.

The following is Mr. Wilson's description of that troublesome disorder affecting the scalp of children, porrigo scutulata or, as Mr. Wilson terms it, trichosis furfuracea.

"In common ringworm, the leading character of the disease is the formation of a thin layer of scurf, either in separate scales around single hairs, or in patches which include several or a considerable number of hairs. In an early stage of this disease, nothing more is perceptible than these scales, but the diagnosis is made evident by the observation that the scurfiness is limited to particular hairs, and clusters of hairs; and that the patches corresponding with these clusters have a circular or oval form. Moreover, the aperture of the follicle of the diseased hair is generally more or less prominent or papillated, having the appearance of being drawn up by the growth of the hair, and bearing a close resemblance to the papillated condition of the skin in 'cutis anserina.' Again, if one of the hairs issuing from this furfuraceous base be gently pulled, the probability is, that it will not come up by the root as in the case of healthy hairs, but will break off near the skin, or within the follicle, according to the period of the disease. The only inward symptom indicative of a disordered action in the scalp is a slight degree of itching, which is relieved as soon as the scurf is torn away by the nails, or removed by the aid of a comb. Indeed, it is this symptom that first makes the parent or guardian aware of the existence of disease.

"The state of skin above described constitutes the first stage of trichosis furfuracea, and may exist for two or three weeks without being particularly noticed. At the end of this time, however, the diseased hairs have grown beyond the follicle, and break off at a short distance from the skin, leaving an uncovered patch of scalp, of variable size, but generally of a circular form. The disease has frequently arrived at its second stage before it is discovered, and before a medical opinion is obtained. The furfuraceous condition of the skin is now very distinct; there is also a slight redness, and sometimes an elevation of the patches above the level of the surrounding surface. The broken hairs are very uneven in point of length, ragged at the ends, and have the appearance of having been eaten through by an insect. This peculiarity has evidently given rise to the term *tinea*, one of the synonyms of the disease; and the same character in conjunction with the circular form of the patches, to the popular appellation *ringworm*.

"The broken stumps of the hairs are also much altered in appearance; they are variously bent and twisted, and lighter in colour than the original hair. In dark-haired children they frequently form little black knobs at the mouths of the follicles; while in light-haired children, the short stumps have an appearance which may be well compared to the fibres of hemp or tow.

"At a later period, if the disease be neglected, the scales collect in large quantities, and become matted together into thick greyish and yellowish crusts. These crusts are thicker and looser at the edges than towards the centre of the patch; and when the latter is large, the crust is broken up into several angular compartments, the line of rupture being remarkable for its white and silvery appearance. Moreover, on the surface of the crust, which is dry and harsh, the tow-like fibres of the diseased hairs may generally be perceived." P. 416.

Under the generic name of Melanopathia Mr. Wilson details some interesting cases of darkening of the skin carried to the extent of almost negro-blackness. And in the Chapter relating to alteration of secretion of the sebiparous glands he explains the occasional production of black secretion by the skin. In an instance of this kind to which he refers, he found, on examining the morbid deposit with the microscope, that it resembled "ordinary sebaceous substance, but the nuclei of the cells, instead of being colourless, were perfectly black and every here and there formed masses of considerable size, indeed they were identical in point of structure with the deepest coloured cells of the rete mucosum of the negro-skin, the nuclei being composed of an aggregation of granules more or less shaded with pigment."

He alludes also to the case published in the Transactions of the Medico-Chirurgical Society, as occurring to Dr. Read of Belfast and Mr. Teevan, and illustrates the subject by the recital of a case of the same kind recorded in an old volume of the Philosophical Transactions.

We have been led by our estimation of this work to take a more extensive review of its contents than is customary in second notices, and we must now conclude by recommending it very warmly to our readers, and especially to the junior members of the profession. They will find the plates supersede the necessity of any others, and the volume to fulfil in every particular the character which it assumes, of being a practical guide to the diagnosis and treatment of diseases of the skin.

